# Supertrees Based on the Subtree Prune-and-Regraft Distance

**DOI:** 10.1093/sysbio/syu023

**Published:** 2014-04-02

**Authors:** Christopher Whidden, Norbert Zeh, Robert G. Beiko

**Affiliations:** Faculty of Computer Science, Dalhousie University, 6050 University Avenue, PO Box 15000, Halifax, Nova Scotia, Canada B3H 4R2

## Abstract

Supertree methods reconcile a set of phylogenetic trees into a single structure that is often interpreted as a branching history of species. A key challenge is combining conflicting evolutionary histories that are due to artifacts of phylogenetic reconstruction and phenomena such as lateral gene transfer (LGT). Many supertree approaches use optimality criteria that do not reflect underlying processes, have known biases, and may be unduly influenced by LGT. We present the first method to construct supertrees by using the subtree prune-and-regraft (SPR) distance as an optimality criterion. Although calculating the rooted SPR distance between a pair of trees is NP-hard, our new maximum agreement forest-based methods can reconcile trees with hundreds of taxa and > 50 transfers in fractions of a second, which enables repeated calculations during the course of an iterative search. Our approach can accommodate trees in which uncertain relationships have been collapsed to multifurcating nodes. Using a series of benchmark datasets simulated under plausible rates of LGT, we show that SPR supertrees are more similar to correct species histories than supertrees based on parsimony or Robinson–Foulds distance criteria. We successfully constructed an SPR supertree from a phylogenomic dataset of 40,631 gene trees that covered 244 genomes representing several major bacterial phyla. Our SPR-based approach also allowed direct inference of highways of gene transfer between bacterial classes and genera. A Small number of these highways connect genera in different phyla and can highlight specific genes implicated in long-distance LGT. [Lateral gene transfer; matrix representation with parsimony; phylogenomics; prokaryotic phylogeny; Robinson–Foulds; subtree prune-and-regraft; supertrees.]

An organism's genome provides a detailed record of its past. However, individual gene trees may be influenced by processes including paralogy and gene loss, lineage sorting, and lateral gene transfer (LGT) (Maddison and Knowles 2006; Galtier and Daubin 2008). Supertree methods generate a single tree that represents the relationships in a set of input trees, which may serve as a hypothesis of organismal descent or relatedness, in most cases by optimizing a similarity criterion. Supertrees have been used to represent large-scale phylogenies including the first phylogeny of nearly all extant mammals (Bininda-Emonds et al. 2007), the first family-level phylogeny of flowering plants (Davies et al. 2004), and the first species-level phylogeny of non-avian dinosaurs (Lloyd et al. 2008). They have also been used to study the extent of LGT in prokaryotes (Beiko et al. 2005) and to disentangle the origin of eukaryotic genomes (Pisani et al. 2007). Supertree methods can take as input sets of gene trees sampled from overlapping but non-identical sets of taxa, in contrast with consensus tree approaches, which require that all input trees contain exactly the same set of leaves. Simulations have shown that supertrees can be more reliable in the presence of a moderate amount of misleading LGT than the supermatrix approach which is based on concatenated alignments of many gene sequences (Lapierre et al. 2012).

Many optimality criteria have been proposed for supertree construction. Matrix representation with parsimony (MRP) (Baum 1992; Ragan 1992) was among the earliest methods proposed and remains the most commonly used, but detailed work with MRP has raised several concerns with the approach. MRP converts input trees into a binary character matrix and finds the most parsimonious tree for this matrix. Although the parsimony problem is NP-hard, fast hill-climbing heuristics in PAUP* or TNT allow MRP to be applied to large data sets (Goloboff 1999; Swofford 2003; Roshan et al. 2004). Although MRP is very effective in practice (Bininda-Emonds and Sanderson 2001; Eulenstein et al. 2004; Chen et al. 2006), it is not clear why the MRP approach performs so well as it can generate relationships that do not belong to any of the source trees (Pisani and Wilkinson 2002) or are contradicted by a majority of source trees (Goloboff 2005) and has problems resulting from its unequal representation of taxa as characters (Purvis 1995). Other supertree methods include consensus supertrees (Adams 1972), majority-rule supertrees (Cotton and Wilkinson 2007), Quartet supertrees (Piaggio-Talice et al. 2004), and Triplet supertrees (Lin et al. 2009). However, supertree methods like MRP that are not based on symmetric tree-to-tree similarity measures may be unduly influenced by the shapes of the input trees (Wilkinson et al. 2005).

Bansal et al. (2010) recently proposed Robinson–Foulds (RF) supertrees, which aim to minimize the total RF distance (Robinson and Foulds 1981) between the supertree and the set of input trees. The RF measure captures the number of bipartitions in one tree that do not exist in another. Fast hill-climbing heuristics make computing rooted RF supertrees feasible from binary input trees. Chaudhary et al. (2012) introduced local search heuristics for constructing RF supertrees from unrooted inputs. While RF appears to be a good criterion for supertrees, it may not be suitable for data sets with substantial amounts of LGT: a single “long-distance” LGT event between distant taxonomic relatives will result in many discordant bipartitions and a large RF distance. If many organisms participate in long-distance LGT, then “phylogenetic compromise” trees (Beiko et al. 2008) may emerge which have a small RF distance yet reflect neither the correct species relationships nor the dominant pathways of gene sharing.

Another well-studied criterion for expressing differences between trees is the subtree prune-and-regraft (SPR) distance (Hein et al. 1996). The SPR operation involves splitting a pendant subtree from the rest of the tree, and reattaching it at a different location, with the rooting of the subtree preserved. The SPR distance is the minimum number of such operations required to reconcile two trees and an SPR supertree minimizes the sum of SPR distances. A single SPR operation can accommodate a long-distance transfer, whereas the RF distance could be drastically increased by such a transfer. We therefore expect that optimizing the SPR distance will be more likely to yield the true tree, as opposed to RF, which may be unduly influenced by large topological dissimilarities in the input trees. The relationship between an SPR operation and the topological consequences of an LGT event (Beiko and Hamilton 2006) makes SPR a natural criterion for assessing a supertree whose constituent trees contain a large number of LGT events. The SPR distance should also treat other sources of discordance in an appropriate way: for example, small differences between trees that arise due to errors of phylogenetic inference would lead to small increases in the SPR distance, the RF distance, or the parsimony score. To date, no SPR-based supertree approach has been developed, in part because computing the SPR distance between two phylogenetic trees is NP-hard (Bordewich and Semple 2005; Hickey et al. 2008).

Combining two recent advances makes SPR supertrees feasible. First, agreement forests (AFs) are sets of subtrees obtained by cutting edges in a pair of trees until no topological disagreement remains; by extension, a maximum AF (MAF) is the AF of two trees obtained by making the fewest possible cuts. The number of trees in an MAF is equivalent to the rooted SPR distance (Bordewich and Semple 2005). Indeed, each edge cut represents a transfer and the proposed series of transfers can be quickly inferred from the MAF (Fig. 1). Whidden and Zeh (2009) and Whidden et al. (2010) developed an algorithm to calculate the MAF of two trees with running time *O*(2.42^*k*^*n*). This result signifies that the worst-case running time of the algorithm depends exponentially on the SPR distance *k* between the two trees, and linearly on the number of leaves *n*. The resulting implementation was orders of magnitude faster than any previous algorithm because the base of the exponent is relatively small, and because the exponent is *k* rather than the typically much larger *n*. New enhancements further improve the running time and allow uncertain relationships to be collapsed into multifurcating nodes.

**Figure 1.**
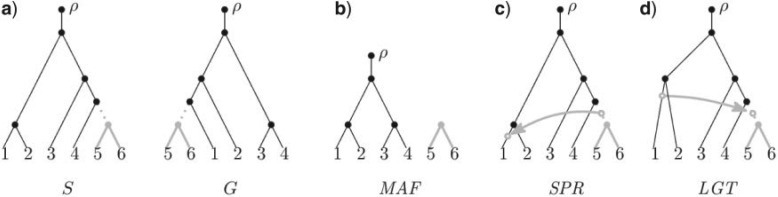
The equivalence between the SPR distance and MAF size. (a) The species tree S and gene tree G differ only in the placement of the gray subtree. The roots of these trees are denoted by *ρ*. (b) The MAF of S and G is produced by cutting the dotted edge in both trees. (c) Each component of an MAF other than the component containing *ρ* represents an SPR move. A single SPR move transforms S into G by moving the gray subtree in S to its position in G. (d) Each SPR move models an LGT event in the reverse direction. From the MAF of S and G we infer that a transfer of gene G has occurred from an ancestor of taxon 1 to an ancestor of taxon 4.

The second advance is a clustering strategy that allows a large problem to be split into smaller, more tractable sub-problems in many cases. Linz and Semple (2011) developed a cluster reduction technique which can reduce the computation of an MAF into several sub-problems, yielding an exponential reduction of the running time in practice. We have adapted this approach and reduced the time required to compute a cluster reduction to linear from the originally published *O*(*n*^3^). By combining the cluster reduction with our improved MAF-based approach, we can process tree pairs that previously required 1–5 h to reconcile in 1 s or less, thus enabling the many SPR distance computations needed to iteratively construct a supertree.

These algorithms are implemented in the SPR Supertree software version 1.2.1, which is available at http://kiwi.cs.dal.ca/Software/SPRSupertrees (last accesed April 2, 2014). The software is freely available, open source, and licensed under the GNU GPL version 3. Here, we describe the steps in our approach and demonstrate the speedups achieved using the algorithmic refinements described above. Our experiments using simulated data sets with LGT show that the SPR approach is more accurate than RF and, for some realistic rates and regimes of LGT, MRP as well. Moreover, we found that MAF-based LGT detection is highly accurate—correctly inferring the exact recipient in 60–80% of inferred transfers. Comparisons based on the eukaryotic data sets used by Bansal et al. (2010) for benchmarking show that the SPR approach yields supertrees with lower total SPR distances to the input trees than either RF or MRP, and with slightly higher RF and parsimony scores. We also used a phylogenomic data set of 244 bacteria to analyze preferential transfer of genes between bacterial lineages. Based on the supertree, we identified putative highways of gene sharing.

## Methods

### Calculating the SPR Distance Between a Pair of Rooted Trees

We can compute the SPR distance between a pair of rooted trees quickly in practice, using a fixed-parameter-bounded search tree algorithm in combination with a linear-time formulation of Linz and Semple's cluster reduction (Linz and Semple 2011) to solve the equivalent MAF problem. Our published MAF algorithm (Whidden et al. 2010; 2013) operates in a bottom-up fashion in the first tree, denoted T_1_, and reduces the second tree to a forest, denoted F_2_. During the algorithm, we identify subtrees that are identical in T_1_ and F_2_ and, in particular, pairs of such trees that are siblings in T_1_ (sibling pairs). If any identical subtree is a component of F_2_, we cut its corresponding parent edge in T_1_. If any sibling pair in T_1_ is also a sibling pair of F_2_, we note that their parent nodes are identical in T_1_ and F_2_. If neither of these two situations applies, we identify at most three possible edge-cutting scenarios and explore each recursively. We explore each scenario in turn, eliminating the need to store a number of searches in memory at once, and use our 3-approximation algorithm (which operates similarly but simply cuts all three possible edges so that its running time scales linearly and may return at most three times the correct distance) to avoid exploring scenarios that cannot be optimal.

We have enhanced our MAF algorithm to prioritize non-branching edge-cut scenarios and ignore duplicate search branches through *edge protection*. First, we examine each sibling pair to select a sibling pair with only one edge-cutting scenario, if any exist. This limits the exponential explosion of our search when possible. Second, we *protect* edges that have been cut in previously rejected scenarios. If we have two scenarios that cut edges *e*_1_ and *e*_2_, respectively, and the *e*_1_ scenario fails to find an MAF, then the *e*_2_ scenario will not find an MAF by cutting *e*_1_ so we *protect* *e*_1_ to indicate this and ignore any scenario that would cut *e*_1_. This prevents us from exploring duplicate edge sets and increases the chance of finding a non-branching edge-cut scenario. When no non-branching sibling pairs remain, we select a sibling pair with a protected member, if possible, to capitalize on this effect. For further details, see online supplementary Appendix I.

We have also extended our MAF algorithm to allow for reconciliation of multifurcating gene trees with the reference supertree (online supplementary Appendix I). For such gene trees, we define the *soft* SPR distance (Linz and Semple 2009; Whidden et al. 2013) to be the minimum number of SPR operations required to transform the reference tree into some binary resolution of the gene tree. This definition accounts for the general assumption that multifurcations imply uncertainty rather than simultaneous speciation. Our algorithm proceeds similarly to the binary case (as the reference tree, required to be T_1_, is binary) with modifications to our considered edge scenarios that allow the resolution of multiple siblings and cutting the resulting edge.

The cluster reduction of Linz and Semple (2011) splits the input trees into smaller sub-problems that can be solved iteratively (but not independently). Two sub-trees of the input trees on the same leaf sets represent a cluster. A cluster MAF with its root edge removed (representing a transfer prior to the LCA of the leaf set) is guaranteed to be part of some complete MAF of the two trees, if any MAF of the two trees cuts this root edge. Alternatively, if every MAF of the cluster must maintain its root edge, every cluster MAF will be part of a complete MAF. We thus modified our search strategy to prefer MAFs with their root edge removed in order to accommodate this reduction. In addition, we removed the complicated weighting scheme of the original cluster reduction method and improved the time required to compute such a cluster reduction to linear in the size of the trees from the cubic scaling reported by Linz and Semple (online supplementary Appendix II).

Recently, Chen and Wang proposed a separate improvement to our previous SPR distance algorithm for binary trees called UltraNet (Chen and Wang 2013). We do not compare our algorithms with UltraNet in detail as UltraNet requires binary trees and failed to find the correct SPR distance in 30 of our 40,631 tests. However, our improved algorithm for the SPR distance even without the cluster reduction was significantly faster than UltraNet and our previous algorithm with clustering outperformed UltraNet on 65 of our tests.

### Supertree Construction

We attempt to find the minimal SPR supertree for a given set of gene trees, that is, a binary rooted tree on the union of the label sets of the gene trees with the minimal cumulative SPR distance to the gene trees (hereafter, simply minimal SPR distance). When the leaf set of the (partially constructed) supertree differs from that of a gene tree, we ignore unique taxa when computing this distance. If no starting tree is provided to initiate the search, we construct an initial SPR supertree through stepwise addition of taxa and then use global SPR rearrangements to optimize the tree. To construct the initial tree, we begin with the four most common taxa in the input trees and select the tree shape on these four taxa with minimal SPR distance to the projected input trees. We then successively add taxa to the supertree, in decreasing order according to the frequency of occurrence in the gene trees. Each taxon is added in the location that minimizes the SPR distance. When determining this location, we only compute the SPR distance to gene trees containing the new taxon, as the SPR distance between the supertree and other gene trees is unchanged. Once we have constructed an initial SPR supertree (or, alternatively, are supplied an initial tree by the user), we begin the SPR rearrangement phase. For a prespecified number of iterations, we look at the *O*(*n*^2^) trees that can be obtained from the current supertree of *n* leaves by one SPR operation and select from these the tree with minimal SPR distance. Many of these SPR rearrangements will be obviously flawed, so we incorporate a bipartition constraint to ignore such rearrangements. Any bipartition of the supertree that is supported by at least half of the gene trees containing two or more taxa from each of the two sets induced by the bipartition is considered “fixed”, and SPR rearrangements that disrupt it are disallowed. This greatly decreases the number of considered rearrangements with little effect on the accuracy of the tree search.

Our methods were developed for rooted gene trees, but we provide three options to accommodate the unrooted gene trees that are typically produced by maximum-likelihood and Bayesian phylogenetic approaches. Our first method is to compute the minimal SPR distance between the supertree and any rooting of each gene tree using an exhaustive search of all possible rootings. Second, given a rooted (partial) supertree and unrooted gene tree, we use each bipartition of the gene tree to try and identify the root bipartition of the supertree. We root the gene tree at the bipartition that best matches the supertree root bipartition according to the balanced accuracy score, an average of the similarities between the matching sides of the bipartitions. Suppose that the supertree root bipartition splits the taxa into two groups A and B and a gene tree bipartition splits the taxa into two groups C and D. Then the balanced accuracy of the C|D bipartition when compared with the A|B bipartition is the larger of (|A ∩ C|/2|A ∪ C|) + (|B ∩ D|/2|B ∪ D|) or (|A ∩ D|/2|A ∪ D|) + (|B ∩ C|/2|B ∪ C|), depending on whether A and C or A and D are more closely matched. Third, we can root the gene trees at a set of outgroup taxa, throwing away trees where this outgroup is not monophyletic. We then build a supertree of this reduced tree set and can then, if desired, root the remainder of the trees using our balanced accuracy approach to build a final supertree.

### Comparative Evaluation and Data Sets

We evaluated the performance of our SPR supertree algorithm against two other approaches: the widely used MRP approach of Baum (1992) and Ragan (1992) and the recently published supertree algorithm of Bansal et al. (2010). Since the RF supertree approach is also based on topological distances between trees, it is an appropriate comparator for our SPR-based method. To construct MRP supertrees, we used the Clann 3.2.2 (Creevey and McInerney 2005) software package to generate matrices for a PAUP* version 4.0b10 (Swofford 2003) parsimony search using 25 iterations of SPR rearrangements (to match the SPR and RF approaches). RF supertrees were constructed using version 2.0 of the software described by Bansal et al. (2010), which uses 25 iterations of SPR rearrangements interleaved with partial data ratchet iterations. In addition to the three basic methods, we tested a variant of SPR supertrees that uses the RF distance as a secondary optimization criterion to break ties when multiple supertrees have the same SPR distance, and tested the SPR and RF supertree methods when the MRP supertree was used as the initial tree. As MRP supertrees are unrooted, we computed the RF and SPR distances for each rooting of the MRP supertree and chose the rooting that gave the minimum value. The three methods were compared in terms of their running time on various data sets as well as their accuracy, either against the known phylogeny in the case of simulated data sets or the three supertree criteria when empirical data sets were used.

To test our supertree approach, we constructed a 244-taxon bacterial SPR supertree from a 40,631-tree subset of the 159,905 unrooted multifurcating prokaryotic phylogenetic trees from Beiko (2011), compared it with an MRP supertree and used the SPR supertree to infer “highways of gene sharing”, that is, frequently implied pathways of LGT among major bacterial lineages. From the 1179 taxa in the original data set, we randomly selected 15 Alphaproteobacteria, Betaproteobacteria and Deltaproteobacteria; 14 Epsilonproteobacteria; 13 Gammaproteobacteria; 40 Bacilli; 34 Clostridia; 74 Actinobacteria; 2 Deferribacteres; 11 Thermotogae; 7 Aquificae; 2 Nitrospira; and 2 Synergistetes for a total of 244 taxa (listed in online Supplementary Table S1, in the Dryad data repository at http://dx.doi.org/10.5061/dryad.h065g) covering a subset of well-sampled and sparsely sampled classes of bacteria and restricted the 159,905 trees to this subset. We then collapsed all branches with a bootstrap support value of less than 0.8 and discarded all star trees and trees with fewer than four taxa. After this procedure, 40,631 trees remained. In total, there were 393,876 leaves in the trees for an average of 9.7 taxa per tree. To construct a supertree from the set of unrooted gene trees, we used our rooting method described above with Aquificae as outgroup. We first constructed an initial guiding supertree from the 40 largest gene trees with a monophyletic Aquificae group (Griffiths and Gupta 2004). This required 13 global rearrangement iterations and 87 CPU hours to converge on a local minimum. The remaining trees were then rooted using our balanced accuracy approach, and we constructed our SPR supertree from this data set using the guiding supertree as a base, which required 16 iterations to converge and 1198 CPU hours.

Once the final supertree was obtained, LGT events were inferred using MAF comparisons between our SPR supertree and the gene trees. We computed a single MAF for each gene tree and determined the equivalent sequence of implied LGT events in less than 1 min. Transfers where both the putative donor and recipient were contained within two distinct genera were counted, and the results visualized as a heatmap and LGT affinity graph constructed using Cytoscape 2.8.3 (Smoot et al. 2011). We ignored directionality as it is often possible to identify partners but not the direction of transfer (Beiko and Ragan 2008). Heatmap values were scaled such that each row had a mean of 0 and standard deviation of 1 and relationships with fewer than 5% of the maximum transfer events for a row or only a single transfer event were filtered out. Two genera were connected by an edge if the number of inferred LGT events between them exceeded 5% of the total number of homologous genes common to at least one member of both genera.

We built simulated data sets to evaluate the accuracy of SPR, MRP, and RF on gene trees generated from a completely known species history. EvolSimulator (Beiko and Charlebois 2007) version 2.2 was used to generate 15 replicated speciation and extinction histories in populations limited to 25 extant genomes. 10,000 simulation iterations were run in all cases. For each of the 15 distinct histories, multiple runs were carried out in which the rate of LGT was varied between 0 (no LGT) and 2.5 events per iteration in increments of 0.1. We also simulated two different LGT regimes: random, in which transfers between any donor/recipient pair were equally probable; and divergence-biased, where donor/recipient exchanges were more likely between closely related genomes (i.e., genomes that share a recent common ancestor), with no LGT at all between genomes that diverged > 5000 generations in the past. The ancestral genome in each simulation (i.e., iteration 1) had 150 genes, and lineages could gain and lose genes to a minimum of 100 and a maximum of 200. A full list of parameter settings can be found in the sample configuration file (see online supplemental material). The resulting gene trees were used to infer supertrees under the SPR, MRP, and RF criteria: supertree accuracy was evaluated based on dissimilarity with the known species tree, and the total distance between the supertree and all gene trees. The accuracy of our MAF-based LGT detection was evaluated using both the known species history and inferred SPR supertree. Comparing LGT events inferred from different histories is a difficult problem, so accuracy was measured by the proportion of inferred events that correctly identified an LGT recipient and transferred gene.

We also compared the three methods using published eukaryotic supertree data sets of marsupials (Cardillo et al. 2004), seabirds (Kennedy et al. 2002), placental mammals (Beck et al. 2006), and papilionoid legumes (Wojciechowski et al. 2000) obtained from http://www.cs.utexas.edu/~phylo/datasets/supertrees.html (last accesed April 2, 2014). These data sets cover between 121 and 558 taxa in 7–726 trees and were used to compare the supertree methods according to their respective supertree optimization criteria, as was done by Bansal et al. (2010).

All supertrees constructed from empirical data, as well as the input bacterial trees we used, are available online as supplementary material from the Dryad data repository at http://dx.doi.org/10.5061/dryad.h065g.

## Results

### Bacterial SPR Supertree and Large-Scale Analysis of LGT

We first present our supertree of 244 bacterial taxa that was constructed from 40,631 unrooted input gene trees using our two-stage outgroup procedure. The taxa selected for our bacterial supertree analysis were chosen to examine several interesting phylogenetic questions in Bacteria. For example, there are two competing hypotheses for the placement of Aquificae. Informational genes such as 16S small-subunit ribosomal RNA suggest that Aquificae are deep-branching and either external to or sister with Thermotogae but the majority of proteins suggest that Aquificae are sister to Epsilonproteobacteria (or other groups such as the Deltaproteobacteria) and not Thermotogae (Boussau et al. 2008). It has been suggested that Aquificae may be closely related to Epsilonproteobacteria with either LGT or a thermophilic G + C bias and long-branch attraction responsible for the observed affinity for Thermotogae (Griffiths and Gupta 2004; Eveleigh et al. 2013). Informational proteins are thought to be transferred infrequently, so it has been more recently suggested that there have been large amounts of LGT between Aquificae and Epsilonproteobacteria (Boussau et al. 2008;. Our data set also includes members of many other groups implicated in LGT, including Deltaproteobacteria and Clostridia: both of these groups show evidence of frequent LGT with other lineages (Dagan et al. 2010; He et al. 2010; Beiko 2011). Other genera frequently associated with high LGT rates including *Pseudomonas* and *Burkholderia* are also included. Finally, several lineages such as Deferribacteres and Synergistetes with relatively few sequenced representatives and an uncertain phylogenetic position (Jumas-Bilak et al. 2009) were included to assess their placements in the SPR supertree.

**Figure 2.**
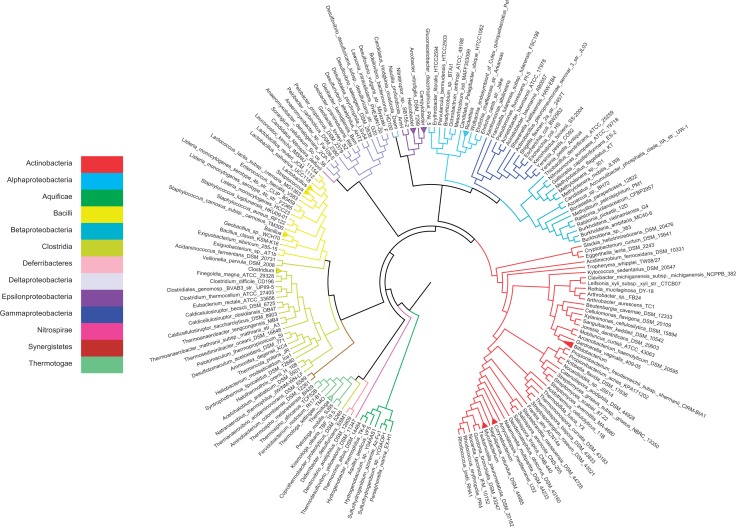
SPR supertree constructed using Aquificae as outgroup. Genera such as *Mycobacterium* with multiple representatives are shown as collapsed subtrees for brevity. Colors indicate the classes of bacteria.

The inferred bacterial SPR supertree (Fig. 2) largely recovered the major bacterial classes as monophyletic groups with several notable exceptions. Deltaproteobacteria are separated from the other Proteobacteria by the Actinobacteria and have a subgroup containing Myxobacteria and *Candidatus* *“Nitrospira defluvii”* (phylum Nitrospirae), for which deltaproteobacterial genomes constitute 7 of the 15 most frequently observed phylogenetic partners. This is an interesting link as both *Candidatus N. defluvii* and *Anaeromyxobacter dehalogenans* are Gram-negative nitrite reducers. Further, it has been suggested that *Ca. N. defluvii* evolved from microaerophilic or even anaerobic ancestors (Lücker et al. 2010) and *A. dehalogenans* exhibits aerobic and anaerobic growth (Sanford et al. 2002).

Among other phylogenetic groups, *Coprothermobacter proteolyticus* shows a particularly interesting affinity, grouping with Thermotagae rather than Clostridia. *Coprothermobacter proteolyticus* was assigned to class Clostridia using small-subunit ribosomal RNA (Rainey and Stackebrandt 1993) but phylogenomic analysis (Beiko 2011; Yutin et al. 2012) and newer phylogenetic trees built from many more samples of small subunit ribosomal RNA agree with a closer relationship between *C. proteolyticus* and Thermotogae (Munoz et al. 2011). With Aquificae as outgroup, the next-deepest branches in the bacterial tree are *Thermodesulfovibrio yellowstonii*, the other member of phylum Nitrospirae, and Deferribacteres, followed by Thermotogae.

**Figure 3.**
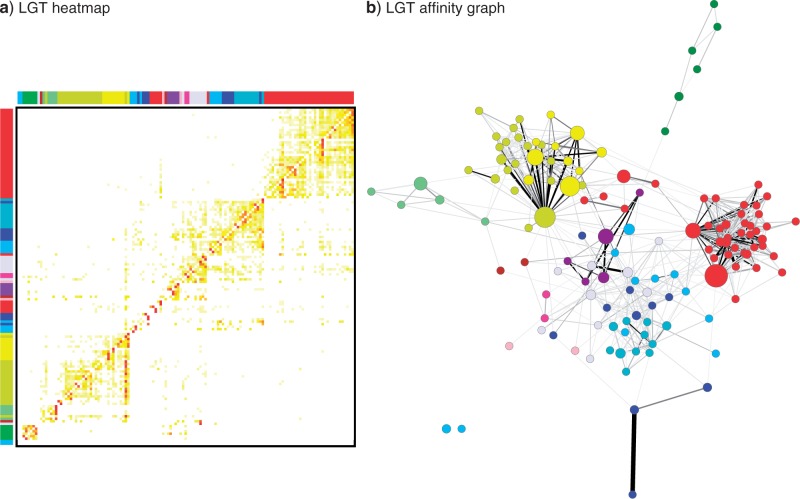
Inferred LGT events between 135 distinct bacterial genera. (a) An LGT heatmap. The colored side bars indicate class using the color mapping of Figure 2. The row and column genus order is the same. The number of transfers is shown in a white-yellow-red color scale with darker colors indicating a higher proportion of transfer events. Color intensity is relative to the largest number of transfers in a row. Relationships with fewer than 5% of the maximum transfer events for a row or only a single transfer event were filtered out. (b) Each node of the LGT affinity graph represents a bacterial genus, colored by class and scaled relative to the number of genomes representing that genus (1–15). Two genera are connected by an edge if the number of inferred LGT events between them exceeds 5% of the number of homologous genes common to both genomes. The shade of an edge is proportional to this ratio of LGT events to common genome size; black edges indicate relationships with at least as many LGT events as the size of their common genome. The thickness of an edge scales relative to the actual number of inferred transfers (between 2 and 370) with thicker edges indicating more transfers. The graph is shown with a spring-loaded layout.

We then inferred LGT events between these bacteria by computing a single MAF for each gene tree and determining the equivalent sequence of implied LGT events. This entire analysis of the 40,631 gene trees required < 1 min using our refined MAF algorithms. Transfer events with source and endpoints both in a monophyletic subtree of the same genus or different genera were identified to focus on relatively recent transfers. Clustering based on the strength of their LGT affinities still groups most genera by class and phylum, and the majority of inferred LGT events occur within clusters of taxonomically related genera (Fig. 3a). In many cases, these relatively small differences between trees are likely to be errors of phylogenetic inference rather than LGT; consequently further evidence (such as association with mobile genetic elements or identification of robust recombination breakpoints) would be necessary to lend further support to the phylogenetic hypothesis of LGT. However, there are also many linkages between genera of distinct phyla and clusters of genera with distinct classes and phyla, which are far less likely to reflect phylogenetic artifacts (Supplementary Fig. S1).

A genus-level LGT affinity graph (Fig. 3b) between genera was used to further explore these relationships and identify paths of gene sharing between distinct lineages. Genera were connected by edges representing transfer events exceeding 5% of their total number of shared homologous genes. As in Figure 3a, the majority of inferred LGT events connect members of the same class or phylum. Yet many linkages connect different classes and phyla such that all of the genera but two, *Ehrlichia* and *Wolbachia*, are connected. The large and diverse genus *Clostridium*, in particular, connects Actinobacteria, Thermotogae, four of the five classes of Proteobacteria, *Thermoanaerovibrio* (phylum Synergistetes), and has many strong connections with Bacilli and other Clostridia (online Supplementary Fig. 2a). Many other inter-phylum connections were observed, especially between specific members of Actinobacteria, Firmicutes, and Proteobacteria. The connectedness of higher taxonomic groups is supported by the class-level affinity graph (online Supplementary Fig. 2b), in which each class is connected to 3.92 other classes on average, with Actinobacteria connected to a total of 10.

### Validation of Efficiency and Accuracy

**Figure 4.**
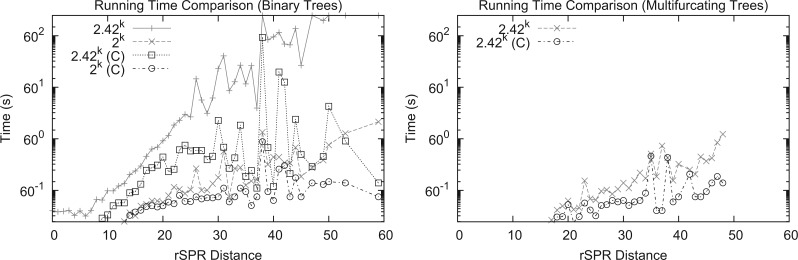
Mean time required to compare gene trees with a given SPR distance from an SPR supertree of a 244-genome data set. The time axis is on a log scale as the time required increases exponentially with the SPR distance. The left panel compares our previous (2.42^*k*^*n*) and new (2^*k*^*n*) algorithms, with (C) and without clustering, on the set of binary trees. The right panel compares our new algorithm with and without clustering on the set of trees with unsupported bipartitions collapsed. Note that collapsing bipartitions reduces the SPR distance.

We next demonstrate the improved performance of our MAF algorithms with a single SPR distance analysis of our 244-taxon bacterial supertree when compared with each of the 40,631 gene trees. Our improved algorithms reduced the time required for individual calculations from 5 h to a maximum of 0.8 s on the initial set of binary gene trees (Fig. 4). Our algorithm requires slightly more time to compare the supertree with multifurcating trees for a given SPR distance but this is balanced by the reduction in SPR distance caused by collapsing unsupported bipartitions; clustered comparisons required at most 0.76 s. As mentioned previously, a full LGT analysis now requires just 34 s on a single CPU. Without our new algorithms, such an analysis would be limited to binary trees and require > 65 h.

### Validation with Simulated Data sets

**Figure 5.**
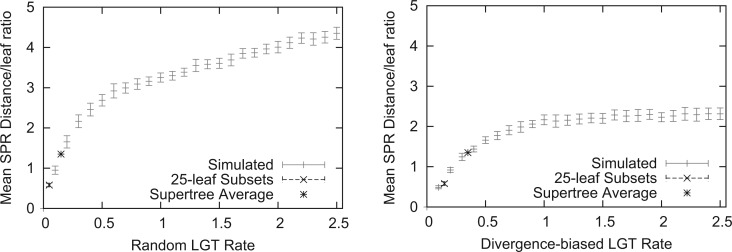
A comparison of our LGT rate simulation parameter to the bacterial data set. Supertrees of empirical data have the same mean SPR distance to leaf ratio (within 95% confidence intervals) as our simulations with a random LGT rate <0.2 and a divergence-biased LGT rate <0.4.

We next compared the ability of SPR, RF, and MRP-based supertrees to recover the species tree in a series of simulated data sets. Simulated LGT rates varied between 0 (no LGT) and 2.5 events per iteration (see the “Methods” section for details). To give context to our LGT rate simulation parameter, we computed the mean ratio of SPR distance to the number of leaves in the simulated trees, to similar values inferred for the 244-taxon SPR supertree (Fig. 5). The inferred frequency of LGT in our empirical data equated to a simulated random LGT rate between 0.1 and 0.2 and a simulated divergence-biased LGT rate between 0.3 and 0.4. Since the bacterial supertree has 244 leaves rather than 25, we also restricted our bacterial supertree and gene trees to 25 randomly sampled subsets of 25 leaves and computed this ratio. We found these sub-sampled supertrees corresponded to lower simulated rates of LGT. This suggests that our simulations with lower rates of LGT are biologically plausible. Although our higher rates exceed the average frequency of LGT, the distribution of LGT events is non-uniform across bacterial lineages (Beiko et al. 2005; Kunin et al. 2005; Thiergart et al. 2012) and our higher simulated rates are likely to be relevant to the inference of some relationships in the supertree.

**Figure 6.**
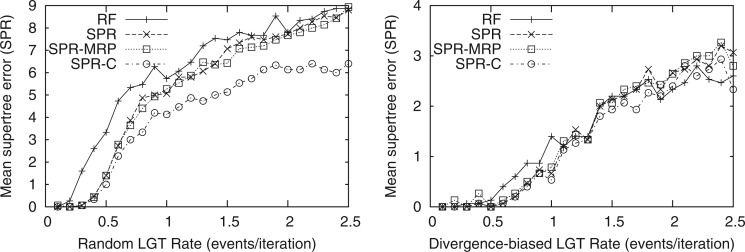
A comparison of the mean supertree error (as measured by the SPR distance) of RF supertrees (RF) to SPR supertrees using the default parameters (SPR), seeded with an MRP starting tree (SPR–MRP), or seeded with the correct tree (SPR-C).

Having established the relevance of our simulated rates of LGT, we then assessed the ability of different supertree algorithms to recover the correct organismal history based on analysis of the gene trees. SPR supertrees were significantly more similar to the simulated species tree than RF supertrees for the LGT rates seen in our bacterial data set and higher (Fig. 6; *P* < 0.05 for random LGT rates of 0.2–0.9, 1.3, and 1.4 and a divergence-biased LGT rate of 1.0 with a two-tailed paired Student's *t*-test; *P* < 0.01 for random LGT rates of 0.2–0.7, 0.9, and 1.4; the overall results were significant with *P* < 10^−5^ for random LGT). Seeding the SPR supertree search with an MRP tree did not substantially change these results. Seeding the SPR supertree search with the correct tree did not substantially change the results for divergence-biased LGT or plausible rates of random LGT, nor did seeding the MRP supertree search with the correct tree. We see that the SPR supertree and the simulated species tree diverge as the random LGT rate increases, even when seeded with the species tree. These results suggest that data sets with substantially higher rates of LGT than our bacterial data would require a better search strategy or a network-based analysis rather than a supertree.

**Figure 7.**
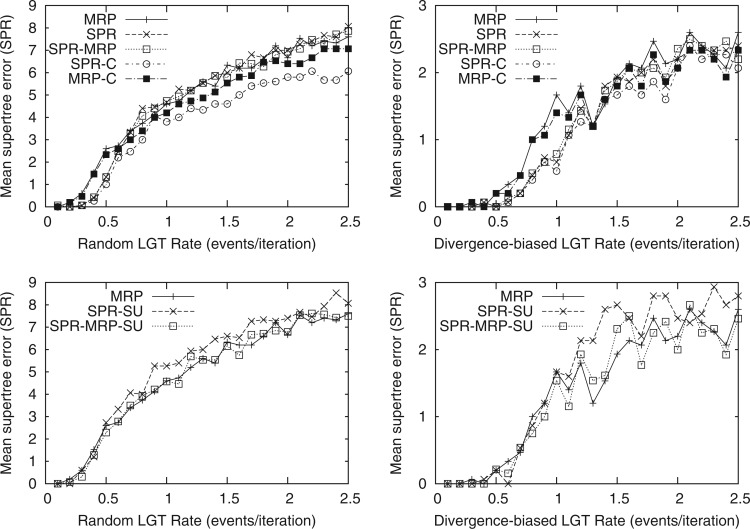
A comparison of the accuracy of SPR and MRP supertrees with known or unknown gene tree roots. The upper panels compare the mean supertree error (as measured by the minimal SPR distance to any rooting of a supertree) when the gene trees are correctly rooted. We compared MRP supertrees using the default parameters (MRP), or seeded with the correct tree (MRP-C) to SPR supertrees using the default parameters (SPR), seeded with an MRP starting tree (SPR–MRP), or seeded with the correct tree (SPR-C). The lower panels compare the mean error of the MRP supertree to SPR supertrees when the gene tree roots are unknown, using our balanced accuracy-based simple unrooted comparison without and with an MRP seed tree (SPR-SU and SPR–MRP-SU, respectively).

As MRP constructs unrooted supertrees, we evaluated accuracy in terms of the minimum SPR distance between the simulated species history and any rooting of the inferred supertrees. The upper panels of Figure 7 show the mean supertree error between the simulated species histories and the MRP supertree, SPR supertree, SPR supertree seeded with an MRP starting tree, and SPR supertree seeded with the correct species tree. The SPR supertrees were significantly more similar to the simulated species history than the MRP trees under biologically plausible rates of LGT (*P* < 0.01 for random LGT rates of 0.3–0.5 with a two-tailed paired Student's *t*-test; the divergence-biased results were not significantly different for individual rates other than 0.6 and 1.0 due to the small supertree error but were significantly better overall with *P* < 0.001). At higher simulated rates of LGT, the accuracy of SPR supertrees matches that of the MRP trees. We observed that this occurs when the accuracy of the SPR supertree and the SPR supertree seeded with the correct tree diverge, suggesting that a better search strategy may improve these results. We also examined the accuracy of RF supertrees with this unrooted measure and found similar results to the unrooted comparison, that is, SPR supertrees and MRP supertrees were both significantly more similar to the simulated species tree than the RF supertrees (online supplementary Fig. S3). The lower panels of Figure 7 show the mean supertree error when gene trees were unrooted. Our balanced accuracy method of rooting was used. The accuracy of our SPR supertrees when the gene tree roots are unknown matches that of the MRP trees for plausible rates of LGT but the performance of our SPR supertrees declines with increasing rates. Using an MRP seed tree prevented this decline, which suggests that our initial tree construction step is not well suited to gene trees with unknown roots. Developing an improved method for building starting trees from unrooted gene trees could improve these results.

**Figure 8.**
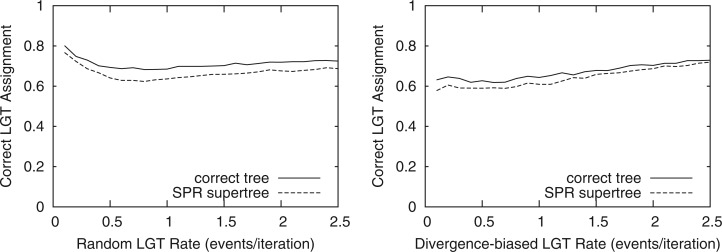
A comparison of the accuracy of MAF-based LGT detection using the correct species history and inferred SPR supertree. Accuracy is measured by the proportion of inferred transfers mapped to the correct gene and LGT recipient.

Having shown the accuracy of SPR supertrees, we next evaluated MAF-based inference of LGT. MAF-based inference was highly accurate for identifying LGT events, identifying an exact LGT recipient in 60–80% of the inferred events on average with a standard error < 0.045 in each case (Fig. 8). Mean accuracy reduced by at most 6.5 percentage points ( < 10%) when using the SPR supertree for inference rather than the correct evolutionary history. Mean accuracy decreased with increasing random LGT rate between 0 and 0.5, whereas a corresponding drop in correct assignment was not seen in the divergence-based set. Above LGT rates of 0.5, accuracy was stable, possibly with a small increase as LGT rates increase. Many other inferred events will identify the target rather than the recipient or map to close relatives of either, justifying our choice to focus on transfers between genera to identify bacterial genesharing.

### Comparison with MRP and RF Supertrees on Eukaryotic Data sets

Bansal et al. (2010) validated their RF supertree approach on a series of eukaryotic data sets that varied substantially in the number of input trees and total number of taxa. We compared the accuracy of each supertree method on the data sets of Bansal et al. (2010) as measured by their ability to minimize the three supertree criteria of SPR distance, RF distance, and parsimony score to the gene trees (Table 1). Each supertree method was best at minimizing its respective optimization measure, suggesting that each method has merit and a well-balanced analysis should either include a justification for the choice of method (e.g., the presence of LGT for the SPR distance) or consider multiple optimization criteria. The MRP method required the least amount of time and the SPR method the most. However, the SPR method converged rapidly in three, one, five, and three iterations on the marsupial, seabird, placental mammal, and legume data sets, respectively, and thus produced an optimal result in only a fraction of the reported time. Seeding the search with the MRP tree greatly reduced the time required by the SPR method and reduced the resulting parsimony scores at the expense of increasing the SPR distance. Starting with the MRP tree reduced the time required by the RF method and found supertrees with better RF and MRP scores on the marsupial and placental mammal data sets but increased RF and MRP scores on the legume data set. Using the RF distance as a tie-breaker with the SPR method found smaller SPR distances, RF distances, and parsimony scores in a shorter period of time than the basic method and avoided an issue with the seabird data set where many supertrees have the same SPR distance but poor RF distances and parsimony scores. These results suggest that blended methods have merit even when only considering a single optimization criterion. In particular, the SPR distance with RF distance as a tie-breaker should be used when non-trivial amounts of LGT are expected.

**Table 1.** Experimental results comparing the performance of the SPR supertree method to RF and MRP supertree methods

Data Set	Supertree Method	SPR Distance	RF-Distance	Parsimony Score	Time (s)
Marsupial (267 taxa; 158 trees)	SPR	382	1604	2203	1097.79
	SPR–RF–TIES	**373**	1536	2149	767.01
	SPR–MRP	380	1534	2126	219.64
	RF-Ratchet	386	1510	2142	688.55
	RF–MRP	381	**1502**	2118	662.95
	MRP–TBR	379	1514	**2112**	**20.52**
Seabirds (121 taxa; 7 trees)	SPR	**17**	109	235	31.15
	SPR–RF–TIES	**17**	63	**208**	29.44
	SPR–MRP	**17**	**61**	**208**	2.04
	RF-Ratchet	**17**	**61**	210	6.34
	RF–MRP	**17**	**61**	209	5.87
	MRP–TBR	**17**	**61**	**208**	**1.03**
Placental mammals (116 taxa; 726 trees)	SPR	1715	5908	8946	5561.84
	SPR–RF–TIES	**1713**	5902	8934	5040.03
	SPR–MRP	**1713**	5876	8921	1819.08
	RF-Ratchet	1784	5718	8830	442.697
	RF–MRP	1781	**5694**	8820	430.77
	MRP–TBR	1783	5702	**8809**	**34.27**
Legumes (558 taxa; 19 trees)	SPR	108	651	1175	21130.08
	SPR–RF–TIES	**92**	471	1037	12376.00
	SPR–MRP	110	511	903	276.49
	RF-Ratchet	126	**409**	1095	403.513
	RF–MRP	136	451	1081	397.62
	MRP–TBR	140	519	**891**	**579.76**

Notes: Six analyses are shown: The SPR supertree method starting from an SPR greedy addition tree (SPR) or MRP supertree (SPR–MRP), the SPR supertree method breaking ties with the RF distance using a greedy addition tree (SPR–RF–TIES), the RF supertree method starting from random addition sequence trees (RF-Ratchet) or MRP supertree (RF–MRP), and MRP with TBR global rearrangements (MRP–TBR). The best optimization criteria or running times for a data set are shown in bold.

### Comparison of SPR and MRP Supertrees of 244 Bacterial Genomes

**Figure 9.**
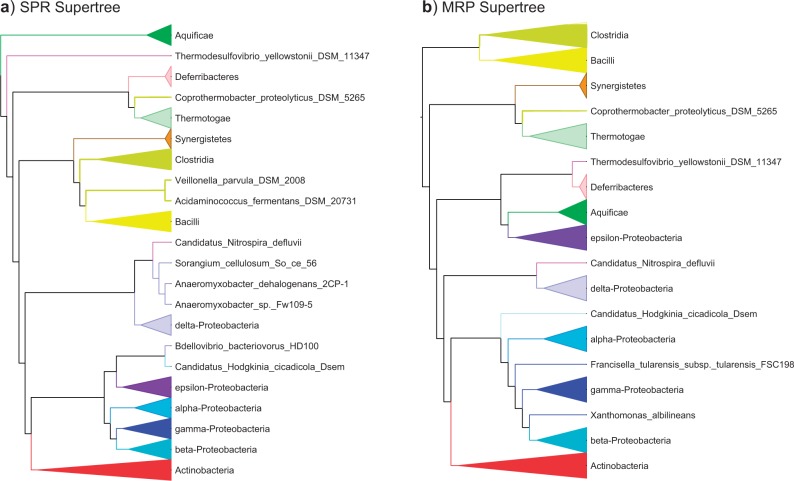
Comparison of SPR and MRP supertrees of 244 bacterial genomes. The SPR supertree on the left was constructed with Aquificae as outgroup while the MRP supertree on the right is unrooted and places Aquificae as neighbors of Epsilonproteobacteria. Both figures show the largest monophyletic group of each class as a collapsed subtree and all members of a given class with the same color.

To contrast with the SPR supertree described above and examine the influence of tree rootings, we constructed an MRP supertree from the 244-taxon bacterial data set using 25 iterations of an SPR rearrangement search and compared it with our SPR supertree (Fig. 9). The MRP supertree does not recover the same arrangement of hyperthermophiles as the SPR supertree; notably, it places the Epsilonproteobacteria in close proximity to Aquificae. If we place the root somewhat arbitrarily between Firmicutes and all other Bacteria, the MRP supertree like the SPR supertree places Thermotogae and *C. proteolyticus* as sisters, although this pairing is sister to Synergistetes and not Deferribacteres as in the MRP supertree. The two Nitrospirae are again split, with *Nitrospira* sister to Deltaproteobacteria and *Thermodesulfovibrio* with Aquficae and Deferribacteres. As with the SPR supertree, Deltaproteobacteria are separated from the other Proteobacteria.

The rooted nature of MAFs allowed the evaluation of our chosen rooting and alternative rootings on inferring phylogenetic relationships from this data set. We have already described the MRP supertree rooted to separate Firmicutes from the other taxa (MRP), the SPR supertree constructed from the 40 largest trees with a monophyletic Aquificae group (40-Aquificae), and the SPR supertree constructed using the SPR-Aquificae supertree (SPR-Aquificae). Three more supertrees were constructed to test the influence of starting topology and rooting. The first was an SPR supertree seeded with the MRP supertree (SPR–MRP). We then rooted the gene trees with both the MRP supertree and SPR-Aquificae tree using our balanced accuracy measure and constructed an SPR supertree from these two sets of rooted gene trees (SPR–MRP-Rooting and SPR-Aquificae-Rooting, respectively).

These six supertrees were compared with the two sets of rooted gene trees (see Table 2). The three MRP-rooted supertrees had a much smaller aggregate SPR distance (nearly 11% smaller) to the MRP-rooted gene trees than Aquificae-rooted supertrees but the three Aquificae-rooted supertrees had a much smaller SPR distance ( > 8% smaller) to Aquificae-rooted gene trees than the three MRP-rooted supertrees. Thus, it is impossible to determine which supertree is more similar to the gene trees without choosing a specific rooting of the gene trees.

**Table 2.** Aggregate SPR distance to supertrees constructed from different rootings of the bacterial protein trees

MRP Rooted Gene Trees	SPR-Aquificae Rooted Gene Trees		
	SPR Distance		SPR Distance
SPR–MRP-Rooting	52,867	SPR-Aquificae-Rooting	53,534
SPR–MRP	52,896	SPR-Aquificae	54,488
MRP	52,896	40-Aquificae	55,570
SPR-Aquificae-Rooting	58,539	SPR–MRP-Rooting	58,023
SPR-Aquificae	59,561	SPR–MRP	58,057
40-Aquificae	60,611	MRP	58,057

Notes: Six different construction methods were compared: The MRP supertree (MRP), the SPR supertree constructed from the 40 largest trees with a monophyletic Aquificae group (40-Aquificae), the SPR supertrees constructed using the MRP supertree (SPR–MRP) or SPR-Aquificae supertree (SPR-Aquificae), and the SPR supertrees constructed by only rooting the gene trees using the MRP supertree (SPR–MRP-Rooting) or SPR-Aquificae tree (SPR-Aquificae-Rooting) and building a greedy addition supertree. Each supertree was compared with the MRP rooted gene trees or SPR-Aquificae rooted gene trees with the SPR distance.

The four SPR supertrees constructed from the full bacterial data set were compared by measuring their pairwise SPR distances (see Table 3). The two Aquificae-rooted supertrees differed by only 10 SPRs, despite the fact that one was constructed from the 40-Aquificae tree and the other was constructed with our usual greedy addition procedure and no *a priori* information other than the gene tree roots. Even more telling, the two MRP-rooted supertrees were essentially identical, differing by only two SPRs. The SPR–MRP-Rooting supertree also differed from the MRP supertree by only two SPRs, so we were able to essentially recover the MRP supertree just by biasing the gene tree roots. This suggests that MRP infers relationships that are consistent with certain gene tree roots despite not implicitly assuming any rooting. As these relationships are also inconsistent with plausible alternative roots, it may be that unrooted supertree methods such as MRP are insufficient to distinguish between controversial evolutionary hypotheses such as the placement of Aquificae.

**Table 3.** Dissimilarity of supertrees constructed from the same rooting of bacterial protein trees

	SPR-Aquificae	SPR-Aquificae-Rooting	SPR–MRP	SPR–MRP-Rooting
SPR-Aquificae	0	10	34	33
SPR-Aquificae-Rooting	10	0	27	25
SPR–MRP	34	27	0	2
SPR–MRP-Rooting	33	25	2	0

Notes: We compared the minimal SPR distance between any rooting of the SPR supertree constructed from the 40 largest trees with a monophyletic Aquificae group (40-Aquificae), the SPR supertrees constructed using the MRP supertree (SPR–MRP) or SPR-Aquificae supertree (SPR-Aquificae), and the SPR supertrees constructed by only rooting the gene trees using the MRP supertree (SPR–MRP-Rooting) or SPR-Aquificae tree (SPR-Aquificae-Rooting) and building a greedy addition supertree.

## Discussion

Using simulations, we verified that SPR supertrees were significantly more similar to the known species history than RF supertrees given biologically plausible rates of simulated LGT. The effect was more pronounced for random LGT, which produces more “long-distance” transfers, than for divergence-biased LGT. These results suggest that penalizing phylogenetic discordance in a manner that is insensitive to the number of impacted bipartitions may be preferable to the alternative RF criterion. However, in the future, this assertion should be tested under a wider range of scenarios, with larger trees and different types of phylogenetic discordance modeled. In particular, our focus on simulated LGT events without considering problems of inference or deep coalescence does not reflect the full spectrum of reasons why trees may disagree. SPR also outperformed MRP in a narrower, but still biologically relevant, range of LGT rates. However, the advantage of SPR disappeared when the gene tree roots were unknown, demonstrating that the obligately rooted SPR approach is influenced by alternative rootings of the reference and gene trees. We also verified that each of the three supertree methods excel at minimizing their respective supertree criteria on a eukaryotic data set. Combining multiple supertree criteria, such as using the RF distance to break ties in an SPR supertree approach, yielded better results than any method did alone. This finding suggests that combinations of criteria that consider different types of phylogenetic discordance may provide even greater accuracy. Furthermore, the SPR approach yielded RF and parsimony scores that were competitive with the RF and MRP approaches on the eukaryotic data sets. Since the majority of phylogenetic discordance in the eukaryotic trees is almost certainly due to factors other than LGT, our results show that SPR is suited to a range of phenomena and not just LGT alone.

Although the history of bacteria may be better represented with a phylogenetic network than a single tree, the supertree we inferred offers a useful backdrop for the inference of highways of gene sharing. Both SPR and MRP recovered a majority of bacterial classes as monophyletic groups, regardless of the choice of rooting, and many of the topological differences between the supertrees were minor. One point of substantial difference between the two trees related to the controversial placement of Aquificae and Epsilonproteobacteria: MRP, being unrooted, placed these two groups adjacent to one another, corresponding to a sister relationship under the reasonable assumption that the root of the supertree is placed somewhere outside of this pairing. When the SPR supertree was constructed from trees rooted to reflect the MRP tree topology in the manner described above, the two supertrees were nearly identical; however, if Aquificae were treated as the outgroup, then the SPR supertree produced a topology that placed other groups with many thermophiles, such as Thermotogae, as early branches. These results suggest that unrooted supertree criteria such as MRP provide hypotheses that are consistent with certain rootings despite not explicitly assuming any rooting. Furthermore, the Aquificae SPR supertree was much more similar to the Aquificae rooted gene trees than the MRP supertree, but the MRP supertree was much more similar to the MRP-rooted trees. It was thus impossible to distinguish between these two hypotheses of Aquificae placement; either could be plausible given knowledge of the correct gene tree roots. This is a practical example of the fundamental limits of unrooted supertree methods identified by Steel and Böcker (2000).

Using the tree in Figure 2 as a basis for LGT inference, we searched for highways of LGT between classes and genera. Not surprisingly, connections were more frequently associated with specific lineages such as *Clostridium* and interactions between Proteobacteria and other phyla varied considerably. In addition, larger gene trees (those shared by many taxa), including trees of ribosomal proteins, required proportionately more transfers to explain, including ribosomal proteins. Such biased LGT could muddy or completely obscure the vertical evolutionary signal. Our improved SPR algorithm allowed the entire set of > 40,000 trees to be reconciled with the supertree in < 1 min: a similar analysis could have been carried out using any rooted reference tree, regardless of what method was used to construct this tree. The rapid inference of LGT highways raises the possibility of using information about lateral connections to construct phylogenetic networks with reticulations explicitly based on major directions of LGT (MacLeod et al. 2005; Nakhleh et al. 2005; Beiko and Hamilton 2006). In a subset of cases, the direction of transfer is unambiguous, which could clarify whether a given highway of gene sharing is unidirectional or bidirectional.

The scaling of running times with the number and size of trees is a central concern in phylogenomics. The analysis of Beiko et al. (2005) required over 20,000 CPU hours to reconcile 22,432 gene trees with a 144-taxon supertree, and the largest trees could not be reconciled at all due to limitations of the breadth-first search of EEEP (Beiko and Hamilton (2006)). Alternative methods of inferring highways of LGT have been proposed based on quartets (Bansal et al. 2013), but such methods are limited to finding the most obvious highways and required on the order of 2 days to analyze the same data set of 22,432 gene trees. Repeated applications of SPR distances in large phylogenomic data sets were heretofore not feasible due to the complexity of the algorithm, but our efficient new methods for computing the SPR distance made the computation of these supertrees feasible even for hundreds of taxa and tens of thousands of gene trees. Of particular importance is the adaptation of the clustering strategy of Linz and Semple (2011) to subdivide the construction of an MAF for a given pair of trees. Clustering yields no improvement in theoretical running time, because there is no guarantee that > 1 cluster will be identified between a pair of trees. However, our results clearly demonstrate that clustering is effective in practice, because LGT connections are not random and consistent clusters can usually be identified. We are optimistic that our approach will be applicable to much larger phylogenomic data sets with thousands of taxa, for two reasons: first, our fixed-parameter algorithm scales exponentially with the *distance* between a pair of trees and not their *size*; and second, as the timing results of Figure 4 suggest, clustering increases the speed of the algorithm and reduces the rate of increase of running times with increasing SPR distance. With only a small number of exceptions, all trees with SPR distance < 60 were resolved in < 1 s, with the time of MAF construction dominated by the single cluster with the largest distance. We expect that most large trees will have a cluster size distribution similar to that of the trees we tested here; consequently, the size of the largest cluster and the corresponding computational burden may increase only slightly. This hypothesis remains to be tested on larger phylogenomic data sets.

In this work, we have focused on comparisons with the MRP and RF supertree approaches. However, many other approaches exist (see, e.g., Bininda-Emonds 2004). Quartet decompositions of trees have shown similar performance to MRP in some studies, although these approaches can be very time-consuming (Swenson et al. 2011). Quartet-based approaches offer an interesting and possible intermediate view of LGT: whereas RF distance can increase substantially from a single “long-distance” LGT event, and SPR treats it as a single topological move, the impact of such an event would be reflected in only a subset of those quartets that contain recipient taxa. A quartet decomposition of a tree contains correlated information, and the effect of this information on supertree inference in the face of LGT and other topological effects is unknown. Another promising set of approaches involves proposing a tree that explicitly reconciles implied duplication, loss, and transfer events given a set of input trees. For example, gene tree parsimony (GTP) aims to minimize evolutionary events that can correspond to duplication and loss, deep coalescence, and LGT events. Supertree approaches generally require single-copy input trees, where no taxon can be represented more than once; by contrast, GTP can accommodate multi-copy trees that arise due to duplication and LGT. GTP and related probabilistic approaches (see, e.g., Heled and Drummond 2010) generally suffer from the same problems of rooting we describe here, and some of the proposed solutions are similar to those we describe above (Chaudhary et al. 2010). GTP and related approaches are also algorithmically complex, and the last few years have seen advances that make these approaches applicable to genome-scale data (e.g., Bansal and Eulenstein 2013). We see supertree and GTP approaches as complementary and view directions that combine our SPR-based optimization strategies, which minimize a simple optimality criterion and yield explicit pathways of discordance, with process-based GTP approaches as a very promising future direction.

Our methods could be expanded and refined in several ways. As we identified in our results, our current supertree search method could potentially be improved with a better strategy for constructing the initial guide tree such as SuperFine (Swenson et al. 2012), methods for avoiding local optima such as ratchet searches, or using prior knowledge to constrain the supertree search (Wehe et al. 2012). An RF supertree method has been recently proposed for multi-labeled gene trees (Chaudhary et al. 2013) and the SPR distance has been defined for such trees by Huber et al. (2011); extending our SPR distance algorithms to accept such trees would enable their inclusion in SPR supertrees. The rooting problem remains to be resolved. While in many cases rooting can be performed using an appropriate outgroup taxon, the bacterial case considered here lacks an obvious outgroup: Archaea could be used to root Bacteria and vice versa, but many gene trees have shown evidence of interdomain LGT and rooting between domains may be invalid or even impossible. Our approach considers only the history of observed genes and does not attempt to account for processes such as gene duplication and loss. Methods of reconciling multiple evolutionary processes such as duplications, losses, transfers, and incomplete lineage sorting show a great deal of promise (Bansal et al. 2012; Szöllosi et al. 2012), but are currently limited to smaller data sets (Stolzer et al. 2012). Finally, the supertree can potentially impose constraints on the timing of LGT events, which can in turn constrain the branching order of the supertree. Such time constraints have been used previously to limit possible transfer scenarios (Beiko and Hamilton 2006; Szöllosi et al. 2012), but phenomena such as donation from extinct lineages and errors of inference must be considered when imposing these constraints (Szöllosi et al. 2013; MacLeod et al. 2005).

## Supplementary Material

Data available from the Dryad Digital Repository: http://dx.doi.org/10.5061/dryad.h065g.

## Funding

The Natural Sciences and Engineering Research Council of Canada via grants to N.Z. and R.G.B., and a graduate fellowship to C.W. C.W. was also supported by the Killam Trusts. N.Z. and R.G.B. acknowledge the support of the Canada Foundation for Innovation and the Canada Research Chairs Program.
